# Gut microbiota as a prognostic biomarker for unresectable hepatocellular carcinoma treated with anti-PD-1 therapy

**DOI:** 10.3389/fgene.2024.1366131

**Published:** 2024-10-03

**Authors:** Yujing Xin, Gang Peng, Wei Song, Xiang Zhou, Xiaoyu Huang, Xiaojing Cao

**Affiliations:** ^1^ Department of Minimally Invasive Comprehensive Treatment of Cancer, Shandong Provincial Hospital Affiliated to Shandong First Medical University, Jinan, Shandong, China; ^2^ National Cancer Center/National Clinical Research Center for Cancer/Cancer Hospital, Chinese Academy of Medical Sciences and Peking Union Medical College, Beijing, China; ^3^ Cancer Hospital, Chinese Academy of Medical Sciences and Peking Union Medical College, Beijing, China

**Keywords:** unresectable hepatocellular carcinoma, gut microbiota, PD-1 inhibitor, lenvatinib, clinical efficacy

## Abstract

**Objective:**

To investigate the relationship between the gut microbiome and the response to anti-PD-1-based combination therapy in unresectable hepatocellular carcinoma (HCC). We aimed to identify potential non-invasive biomarkers and new strategies to modulate immunotherapy in HCC.

**Methods:**

In this study, fresh stool samples and clinical data were collected from unresectable HCC patients treated with anti-PD-1-based combination therapy at the Cancer Hospital of the Chinese Academy of Medical Sciences between January 2020 and December 2021. The patients were divided into two groups based on their response to treatment: the treatment responder group (R group) and the treatment non-responder group (NR group). The composition and diversity of the gut microbiome were bioinformatically analyzed by using the Whole Genome Shotgun strategy, including taxonomic composition analysis, Alpha diversity analysis, Beta diversity analysis, and differentially enriched bacterial taxa analysis. Differentially enriched bacterial taxa between R and NR groups were identified based on the magnitude of the linear discriminant analysis effect size (LEfSe) and analyzed for their impact on the survival of the patient.

**Results:**

A total of 45 eligible patients with unresectable HCC treated with anti-PD-1-based combination therapy participated in this study. The gut microbiological composition and Alpha diversity of patients were not statistically different, but there was a statistically significant difference in Beta diversity between the R and NR groups. (PERMANOVA tests, P = 0.006). We further identified 56 enriched bacterial taxa in the R group and 44 enriched bacterial taxa in the NR group based on the LEfSe analysis (LDA >2.66, P< 0.05). Patients with a high abundance of Collinsella genus, Ruminococcus_AM4211, and Ruminococcus_AF25_28AC had a longer median PFS and median OS compared to those with low abundance (P < 0.05). On the contrary, the median PFS and OS of patients with a high abundance of *Bacteroides*_AF20_13LB and Veillonella_atypica were significantly shorter than those of patients with low abundance (P < 0.05). The multivariate analysis showed that the abundance of *Bacteroides*_AF20_13LB and Ruminococcus_ AF25_28AC was independent related factors for PFS, and the abundance of *Bacteroides*_AF20_13LB was an independent related factor of OS.

**Conclusion:**

The enrichment of specific gut microbiota affected clinical efficacy and survival benefits in HCC treated with anti-PD-1 therapy and may be a promising non-invasive gut microbial biomarker and a new strategy for modulating immunotherapy in HCC.

## Introduction

Hepatocellular carcinoma (HCC) is the most common primary liver malignancy, ranking sixth globally in morbidity and third in mortality in 2020 (Sung et al., 2021). Surgical resection, liver transplantation, or ablation therapy can provide good prognoses for patients with early-stage HCC. Unfortunately, patients with unresectable HCC, particularly those with high tumor burden, have limited survival benefits due to significant tumor heterogeneity (Villanueva, 2019; Bruix et al., 2005).

The continuous advancements of molecularly targeted therapy and immunotherapy are changing the treatment landscape for HCC (Llovet et al., 2018; Llovet et al., 2022). Traditionally, sorafenib or Lenvatinib as first-line tyrosinase inhibitors (TKIs), and Cabozantinib, regorafenib, or ramucirumab as second-line TKIs can modestly prolong the survival of advanced HCC patients (Benson et al., 2021). Immune-checkpoint inhibitors (ICIs) including pembrolizumab or nivolumab have exhibited promising antitumor immunoactivity for HCC in previous clinical trials (Sangro et al., 2021; Zhu et al., 2018; Yau et al., 2022; Sangro et al., 2013; Yau et al., 2020).Atezolizumab plus bevacizumab was approved as the first first-line combination therapy of unresectable HCC with positive results in the IMbrave150 trial (Finn et al., 2020a), and other PD-1 inhibitors combined with TKIs, such as Sintilimab plus bevacizumab biosimilar (Ren et al., 2021), pembrolizumab plus lenvatinib (Finn et al., 2020b), Camrelizumab plus apatinib (Xu et al., 2021), also demonstrated favorable therapeutic effects and tolerable toxicity in current clinical trials. TKIs can reverse the immunosuppressive microenvironment of tumors and enhance the immune anti-tumor efficacy of PD-1 inhibitors, which may provide a rationale for combination therapy (Llovet et al., 2022; Huinen et al., 2021). However, due to the heterogeneity and complex etiology of HCC, patients respond differently to immunotherapy, and there are still some patients who do not benefit from immunotherapy. Therefore, exploring new therapeutic targets and finding biomarkers to predict response to immunotherapy is essential to optimize therapy and guide clinical decisions.

Exploration of biomarkers of immunotherapy has been a research hotspot in cancer immunotherapy. Current biomarker studies on immunotherapy of HCC mainly include tumor genomic features, PD-L1 expression, tumor mutation burden, and microsatellite instability (Harding et al., 2019; Muhammed et al., 2022). However, the use of these indicators in clinical practice is limited due to invasive operations and high costs. A growing number of clinical studies demonstrated that gut microbiota can serve as a potential diagnostic or predictive tool and a novel therapeutic target for HCC. The gut microbiota plays a key role in the development of HCC and anticancer immune responses (Sepich-Poore et al., 2021; Gopalakrishnan et al., 2018a). Gut microbial dysbiosis and leaky gut are prominent features of chronic liver disease and promote the progressive development of chronic hepatitis to liver fibrosis, cirrhosis, and HCC (Schwabe and Greten, 2020). In addition, recent studies have found an important role of gut microbes in regulating the therapeutic response of ICIs, and the changes in the gut microbiome can improve the anti-tumor efficacy of PD-1 inhibitors in patients with non-small cell lung cancer, renal cell carcinoma, and melanoma (Gopalakrishnan et al., 2018b; Ting et al., 2022; Lu et al., 2022). Previous studies have reported significant differences in the gut microbiome among responders in HCC patients treated with PD-1 inhibitors, suggesting that changes in the gut microbiome are associated with response to immunotherapy in HCC (Mao et al., 2021; Lee et al., 2022; Zheng et al., 2019).

To date, the correlation between the gut microbiome and response and survival of HCC patients receiving ICIs has not been fully elucidated, and the value of gut microbiome characteristics in the clinical efficacy of receiving anti-PD-1-based combination therapy for HCC has not been investigated. Therefore, this study used metagenomic sequencing to analyze the stool samples of primary HCC patients treated with anti-PD-1 based combination therapy, aiming to explore the correlation between the diversity and abundance of gut microbes and treatment response and survival prognosis and to find potential non-invasive biomarkers and new strategies to modulate immunotherapy in HCC.

## Methods

### Study population

Between January 2020 to December 2021, patients diagnosed with unresectable HCC who had not received prior treatment from the National Cancer Center were included in this study. After consultation by a multidisciplinary medical team consisting of hepatobiliary surgery, interventional therapy, and medical oncology. The anti-PD-1-based combination therapy of TACE, Lenvatinib, and PD-1 inhibitor was recommended as a treatment option due to excellent antitumor activity. All patients were informed of the potential efficacy, adverse effects, and medical costs of combination therapy. The choice of treatment was ultimately made by the patient. This study was approved by the Institutional Review Boards of the National Cancer Center, Written informed consent for the treatment was obtained from each patient. The need for written informed consent to publish the data was waived by the Ethics Committees, since the personal details of these patients were kept confidential.

The criteria for eligible patients were as follows: ≥18 years old, unresectable HCC diagnosed as Barcelona Clinic Liver Cancer (BCLC) B or C stage by clinical guidelines (Heimbach et al., 2018; European Association for the Study of the Liver. Electronic address: easloffice@easloffice.euEuropean Association for the Study of the Liver, 2018b), liver function was rated as Child-Pugh A class, Eastern Cooperative Oncology Group Performance Status score (ECOG PS) of 0–1, at least one measurable target lesion that can be assessed by modified Response Evaluation Criteria in Solid Tumors (mRECIST) (Llovet and Lencioni, 2020), appropriate organ and hematologic function, the patient has no diarrhea, constipation, and special eating habits, such as long-term use of probiotics, antibiotics or other medications, single foods, and irregular eating habits.

The exclusion criteria included the following: Patient has taken antibiotics, yogurt, probiotics, prebiotics, and proton pump inhibitors within 1 month before treatment; history of inflammatory bowel disease or irritable bowel syndrome; patients who have received previous locoregional or systemic therapy; symptomatic brain metastasis, other malignant tumors, active autoimmune disease, incomplete medical information, and loss of follow-up.

### Treatment procedure

#### Lenvatinib and PD-1 inhibitor administration

Patients received 8 mg (bodyweight < 60 kg) or 12 mg (bodyweight ≥ 60 kg) of Lenvatinib orally once a day, and intravenously every 3 weeks with PD-1 inhibitor including sintilimab (Innovent Biologics, Suzhou, China), tislelizumab (BeiGene, Shanghai, China) or camrelizumab (Hengrui Pharma, Lianyungang, China). Disease progression and intolerable toxicity led to the interruption of treatment or dose adjustment.

#### The procedure of super-selective conventional TACE (cTACE)

The operation of TACE was completed by a senior interventional physician with 15 years of clinical experience. Firstly, the femoral artery was percutaneously punctured using Seldinger’s technique. Then, the 5 French catheters were inserted into the celiac trunk or superior mesenteric artery for arteriography, and a 2.7 French microcatheter was super selectively placed into the feeding arteries of the tumor. Oxaliplatin (75 mg/m2) and raltitrexed (3 mg/m^2^) or 5-fluorouracil (750 mg/m^2^) were slowly delivered into tumor arteries via microcatheter. Then, the emulsion of iodized oil (10–20 mL) mixed with chemotherapeutic drugs (epirubicin,30–50 mg/m^2^ or pirarubicin,20 mg/m^2^) was delivered into the tumor-feeding artery. Last, the feeding artery of the tumor was completely embolized with gelatin sponge particles. The specific dosage of chemotherapeutic drugs and lipiodol was determined by a comprehensive analysis of the patient’s body surface area, tumor size, location, and liver function score. TACE was repeated as needed when residual tumors were found during routine postoperative reexamination.

### Data collection and follow-up

The clinical baseline characteristics and follow-up data of eligible patients were collected and analyzed through medical records, including sex, age, cirrhosis, ECOG PS score, etiology, history of antiviral therapy, maximum tumor size, tumor number, α-fetoprotein (AFP) level, alanine aminotransferase (ALT), albumin-bilirubin (ALBI) grade, albumin (ALB), aspartate aminotransferase (AST), total bilirubin (TBIL). Cirrhosis was diagnosed by medical imaging (e.g., ultrasonography, CT, MRI elastography), liver function tests (e.g., AST, ALT), and etiology (e.g., HBV, HCV) according to liver cirrhosis guidelines of EASL (European Association for the Study of the Liver. Electronic address: easloffice@easloffice.euEuropean Association for the Study of the Liver, 2018a). Imaging evaluation and review by experienced radiologists. The patient’s follow-up data were recorded every 2 months until the disease progression or death. The last follow-up of this study was on 31 December 2022.

### Outcomes and assessments

The main endpoints of this study included overall survival (OS), progression-free survival (PFS), and optimal objective response rate (ORR) based on mRECIST. OS referred to the time interval from initial combination therapy to death from any cause. PFS referred to the time interval from the beginning of the initial combination therapy to the progression of the disease or death. Tumor response was assessed by contrast-enhanced MR or CT every 4–8 weeks and classified as progressive disease (PD), stable disease (SD), partial response (PR), and complete response (CR) based on mRECIST. The “treatment response” in this study referred to the optimal tumor response among all tumor evaluation time points. The patients were divided into two groups according to the optimal tumor response, of which the patients with CR and PR were the treatment responder group (R group), and the patients with SD and PD were the treatment non-responder group (NR group).

### Processing and analysis of gut microbiome genomic data

#### Collection and processing of fecal samples

Fresh stool specimens were collected from patients within 3 days before and after treatment using sterile containers and immediately frozen in a −80° refrigerator, followed by extraction of bacterial DNA from the samples for quality testing.

#### Whole-genome shotgun (WGS) strategy

This analysis of the study was based on the Illumina NovaSeq/HiSeq high-throughput sequencing platform, adopted the whole-genome shotgun (WGS) strategy to randomly break the extracted total genomic DNA of the gut microbiome into short fragments, and constructed the inserted fragment library with appropriate length, and performed double-end sequencing on this libraries (Venter et al., 1998). Dual-end sequence raw data from high-throughput sequencing machines were screened and filtered to remove non-target sequences and obtain high-quality datasets that are available for metagenomic analysis. Next, the species annotation of the gut microbial gene sequences was performed using Kraken2. By counting the bacterial taxa annotation information, the species composition and abundance of each sample in each taxonomic level (phylum, class, order, order, family, genus, and species) can be obtained (Wood et al., 2019).

In order to ensure the quality and reliability of subsequent analyses, it is necessary to screen and filter the raw data from the sequencing machine. The screening and filtering process of raw sequence data mainly includes the following steps: First. Use Cutadapt (v1.17) to identify potential splice sequences at the 3′ end (rarely occurs with passages) and truncate at the identified splice sequences. Match length to the splice sequence was required to be at least 3 bp in length and allow for up to 20% base mismatch rate. Secondly, after removing the 3′ end junction sequence, the sequence was screened for quality using fastp (v0.20.0) using the sliding window method: the window size was 5 bp, starting from the first base position at the 5′ end, requiring that the average quality of the bases in the window was greater than or equal to Q20 (i.e., the average base-averaged sequencing accuracy was greater than 99%), and the sequence was truncated at the 3′ end of the window from the first window that had an average quality value lower than Q20. The sequence is truncated at the 3′ end of the window from the first base with an average quality value lower than Q20.Sequences less than 50 bp in length and sequences containing ambiguous bases in the sequence were removed after the quality screen described above. After the above processing, a clean data set is obtained that can be used for subsequent analyses.

#### Bioinformatics analysis

Bioinformatics analysis of the composition and diversity of the patient’s fecal microbiota was performed with QIIME 2 software (Bolyen et al., 2019) and R software (version 3.6.2), focusing on taxonomic composition analysis, Alpha diversity analysis, Beta diversity analysis, and differential bacterial analysis. Alpha diversity was analyzed by the Chao1 index, ACE index, Shannon index, and Simpson index. Chao1 index and ACE index focused on reflecting the richness of fecal microbiota, while the Shannon index and Simpson index focused on reflecting the evenness of fecal microbiota. The species-level compositional profiles of all samples were first randomly re-sampled at the lowest sequencing depth, so as to correct for the diversity differences caused by sequencing depth. Subsequently, the QIIME software was used to calculate the four diversity indices for each sample. Beta diversity analysis is based on the principal coordinate analysis (PCoA) of microbiota, which was measured by Bray-Curtis dissimilarity and compared by permutational multivariate analysis of variance (PERMANOVA) test. The Adonis/PERMANOVA analysis evaluates the magnitude and statistical significance of the differences between groups of the original samples by comparing the differences between groups of the original samples with the distance matrix obtained by randomly substituting the distances several times and then performing a permutation test on the species distance matrix, which is analyzed by using the QIIME software and performing a 999 times permutation test to determine whether the differences between groups are statistically significant or not. For the differentiated species analysis, the metagenomeSeq (Paulson et al., 2013) method was used as the default method for differential species analysis in this study, whereby taxonomic units at the phylum, genus and species level were compared two by two to obtain the results of the statistical analysis of significance of differences, and the Benjamini-Hochberg method was used to control for the false discovery rate (FDR), and the taxonomic units with a P < 0.05 were selected as species with significant differences in abundance for the subsequent mapping analysis (heat map analysis of differential species clustering).Linear discriminant analysis (LDA) effect size (LEfSe) analysis was performed with an α value of 0.05 (by Kruskal Wallis and Wilcoxon rank-sum tests) and an effect size threshold of 2.66 for LDA to determine the most likely candidate taxa that could explain the differences between groups (Quast et al., 2013). The Whole-shotgun metagenomics data of this study has been publicly available in the NCBI SRA database (PRJNA1142390).

### Statistical analysis

Baseline characteristics and response rates were expressed in terms of frequencies and percentages, and variables were indicated as either the mean (range) or median (standard deviation). Categorical and continuous variables were analyzed with chi-square and t-tests, respectively. Between-group differences in patients’ clinical characteristics were compared by chi-square test or Fisher’s exact test. Differences in species abundance between subgroups at different species levels were compared using the Mann-Whitney U test. After obtaining species that differed significantly across subgroups, the mean value of relative species abundance was used as the cut-off value to classify patients into high and low abundance groups. Clinical factors that significantly affected the distribution of the sample or the distribution of differential species were screened by redundancy analysis (RDA) using a permutation test. The Kaplan–Meier method was used to estimate OS and PFS, and univariate and multivariate regression analyses were used to analyze the prognostic factors of OS and PFS. Data analysis and graphical visualization were implemented using R software (version 3.6.2). P < 0.05 was considered statistically significant.

## Results

### Baseline characteristics of the patients

Between January 2020 and December 2021, a total of 45 eligible primary HCC patients were included in this study. The baseline characteristics were shown in Table 1. The patients in this study were diagnosed with unresectable intermediate-advanced HCC with high tumor burden, and the average size of the maximum tumor was 8.9 cm. The mean age of overall patients was 55.8 years, with 88.9% of patients with Child-Pugh A liver function. Forty-three patients (95.6%) had HBV infections and received antiviral therapy.

**TABLE 1:** Patient demographics and clinical characteristics.

Characteristics	Total (n = 45)	R group (n = 30)	NR group (n = 15)	P value
Sex				0.890
Male	37 (82.2)	24 (80.0)	13 (86.7)	
Female	8 (17.8)	6 (20.0)	2 (13.3)	
Age, years				0.587
<60	28 (62.2)	20 (66.7)	8 (53.3)	
≥60	17 (37.8)	10 (33.3)	7 (46.7)	
ECOG-PS				0.526
0	42 (93.3)	29 (96.7)	13 (86.7)	
1	3 (6.7)	1 (3.3)	2 (13.3)	
Hepatitis B				0.798
Yes	43 (95.6)	28 (93.3)	15 (100.0)	
No	2 (4.4)	2 (6.7)	0 (0.0)	
Cirrhosis				0.916
Yes	22 (48.9)	14 (46.7)	8 (53.3)	
No	23 (51.1)	16 (53.3)	7 (46.7)	
Child-Pugh grade				0.867
A	40 (88.9)	27 (90.0)	13 (86.7)	
B	5 (11.1)	3 (10.0)	2 (13.3)	
Tumor size (mean, cm)	8.8	8.6	9.5	0.333
<10	27 (60.0)	20 (66.7)	7 (46.7)	
≥10	18 (40.0)	10 (33.3)	8 (53.3)	
Tumor distribution				
Single lobe	17 (37.8)	12 (40.0)	5 (33.3)	
double lobe	28 (62.2)	18 (60.0)	10 (66.7)	
PVTT				0.342
Yes	21 (46.7)	12 (40.0)	9 (60.0)	
No	24 (53.3)	18 (60.0)	6 (40.0)	
Extrahepatic metastasis				0.907
Yes	13 (28.9)	9 (30.0)	4 (26.7)	
No	32 (71.1)	21 (70.0)	11 (73.3)	
BCLC stage				0.915
B	19 (42.2)	13 (43.3)	6 (40.0)	
C	26 (57.8)	17 (56.7)	9 (60.0)	
AFP (ng/mL)				0.721
<400	12 (26.7)	9 (30.0)	3 (20.0)	
≥400	33 (73.3)	21 (70.0)	12 (80.0)	
AST (U/L)				0.912
<40	16 (35.6)	10 (33.3)	6 (40.0)	
≥40	29 (64.4)	20 (66.7)	9 (60.0)	
ALT ((U/L)				0.916
<40	20 (44.4)	13 (43.3)	7 (46.7)	
≥40	25 (55.6)	17 (56.7)	8 (53.3)	

Data were presented as n (%) or mean ± standard deviation. Abbreviations: R, treatment responder; NR, treatment non-responder; ECOG-PS, eastern cooperative oncology group performance status; PVTT, portal vein tumor thrombus; BCLC, barcelona clinic liver cancer; AFP, alpha-fetoprotein; ALT, alanine aminotransferase; AST, aspartate aminotransferase.

The optimal tumor response rate was evaluated according to the mRECIST standard. Four patients were evaluated as CR, 26 patients were evaluated as PR, seven patients were evaluated as SD, and eight patients were evaluated as PD. The ORR and DCR for patients were 66.7% and 82.2%, respectively. The patients were divided into two groups according to the treatment response, of which the patients with CR and PR were the treatment responder group (R group), and the patients with SD and PD were the treatment non-responder group (NR group). As shown in Table 1, there were no statistical differences in gender, age, tumor size, number, liver function, and BCLC stage between the R and NR groups.

**FIGURE 1:**
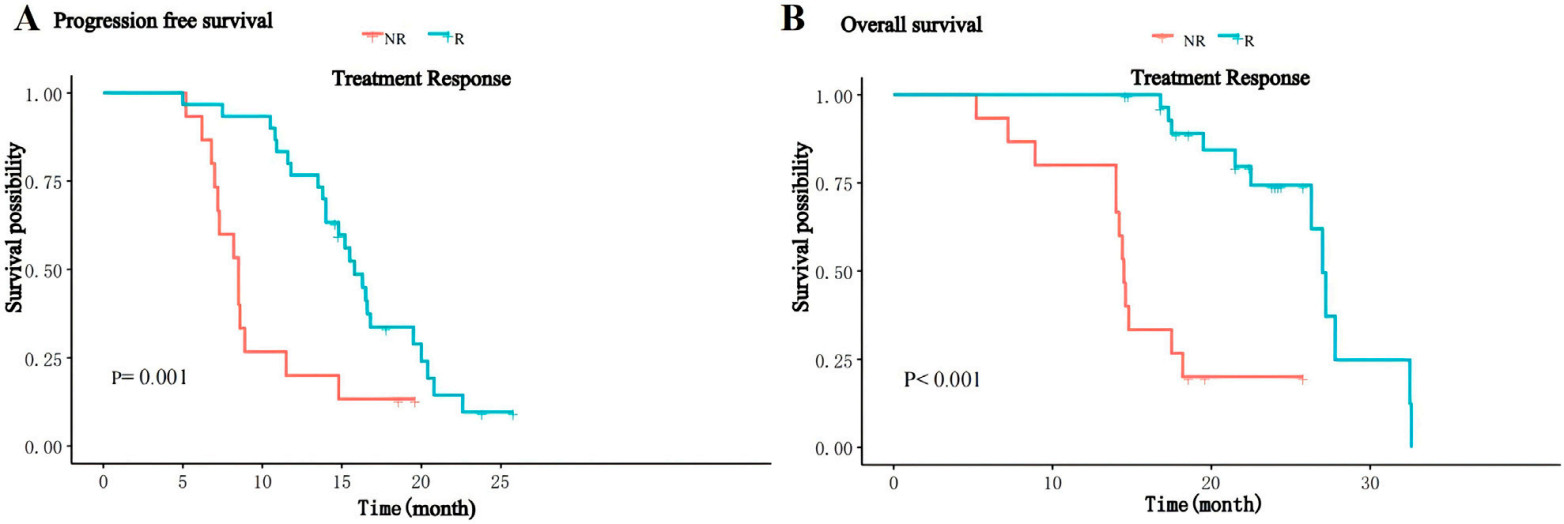
Kaplan-Meier curves for PFS **(A)** and OS **(B)** of patients between the R and NR groups.

The median follow-up of patients was 18.2 (range 5.2–32.6) months, and by the end of follow-up, 37 (82.2%) patients had progressive disease, of whom eight received local radiotherapy, 12 received other immune checkpoint inhibitors and 17 received second-line molecularly targeted drugs (sorafenib or regorafenib), and 24 (53.3%) patients died. The median PFS of the overall patients was 14.0 (IQR: 8.6–16.8) months, and the median OS was 26.3 (IQR:14.8–24) months. The median PFS in the R group was 15.8 (IQR:13.6–17.8) months, significantly higher than 8.0 (IQR:7.1–10.2) months in the NR group (P = 0.001, Figure 1A); the median OS in the R group was 27.0(IQR:17.8–24.5) months, significantly higher than 14.5(IQR:14.0–17.9) months in the NR group (P < 0.001, Figure 1B). Thirty-seven patients in this study experienced disease progression and discontinued treatment and subsequently received second-line therapy, and five patients had dose reductions for grade 3–4 adverse reactions.

### Association of treatment response with gut microbial composition

**FIGURE 2:**
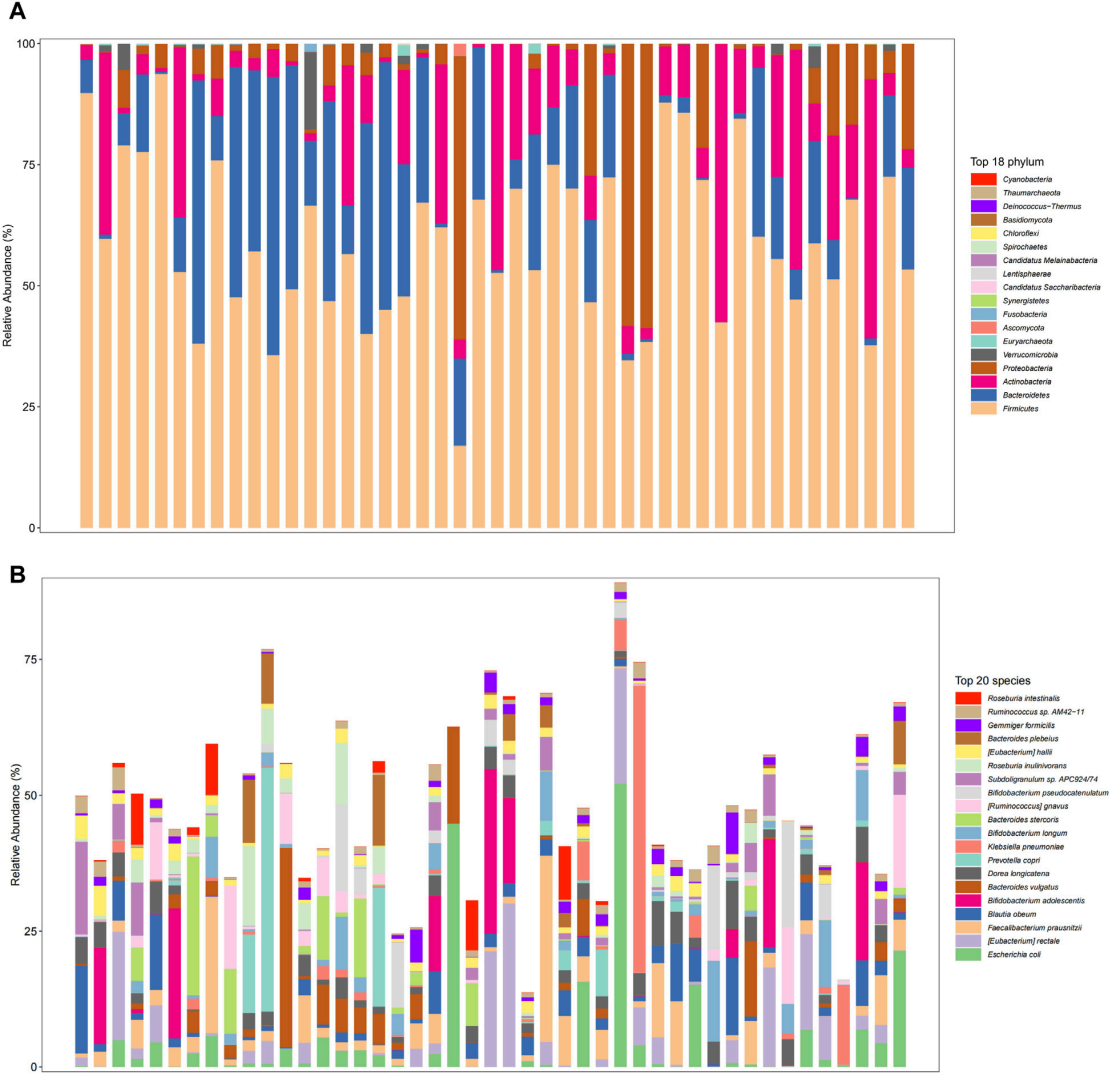
Gut microbiome composition at the phylum level (**(A)**, Top 18 phylum) and species level (**(B)**, Top 20 species) in total patients.

The gut microbiome in the overall fecal samples was classified into 18 phyla, 31 classes, 62 orders, 128 families, 374 genera, and 2092 species of the bacterial kingdom. In the overall sample, at the phylum level (Figure 2A) the relative abundance of the gut microbial communities was the Firmicutes (59.2%), Bacteroidetes (17.8%), Actinobacteria (13.6%), Proteobacteria (8.3%), and Verrucomicrobia (0.8%) in descending order. At the species level (Figure 2B), the relative abundance of gut microbial communities was *Escherichia coli* (4.8%), Faecalibacterium Prausnitzii (4.3%), Blautia obeum (3.3%), Bifidobacterium adolescentis (3.2%) and *Bacteroides* vulgatus (3.0%) in descending order.

**FIGURE 3:**
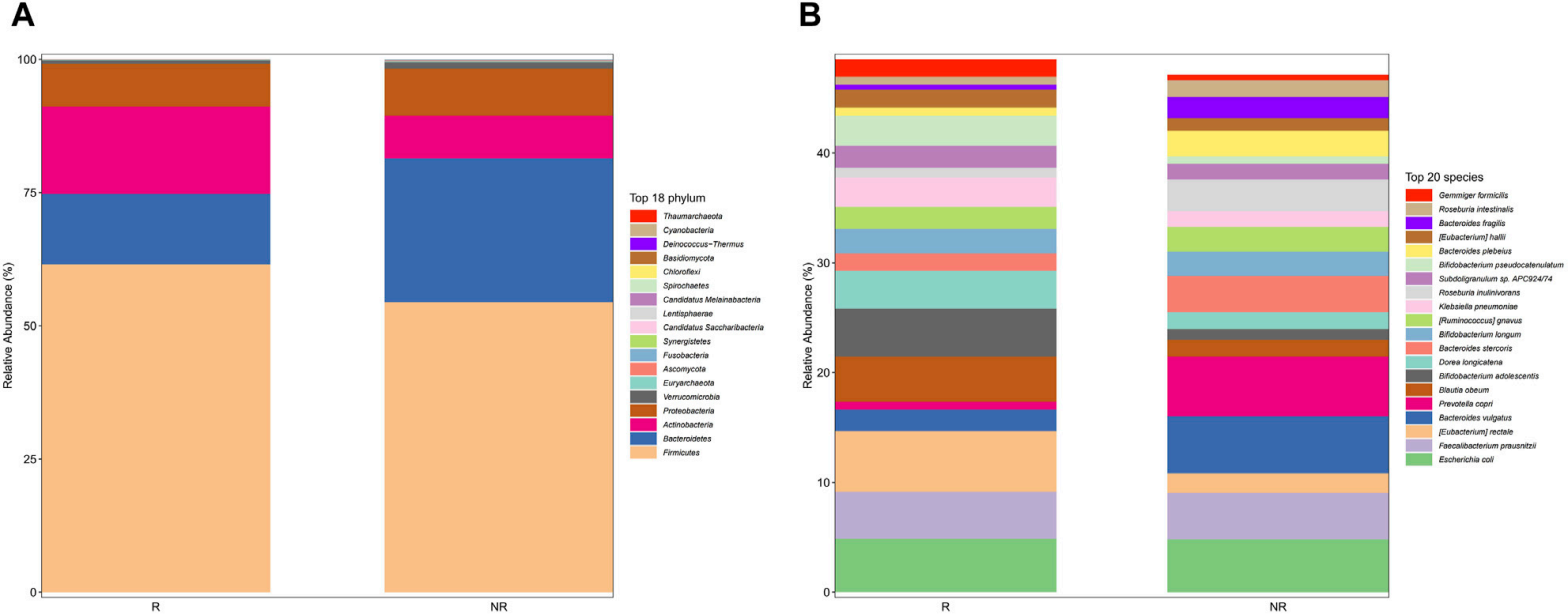
Gut microbiome composition at phylum **(A)** and species **(B)** in the R and NR groups.

**FIGURE 4:**
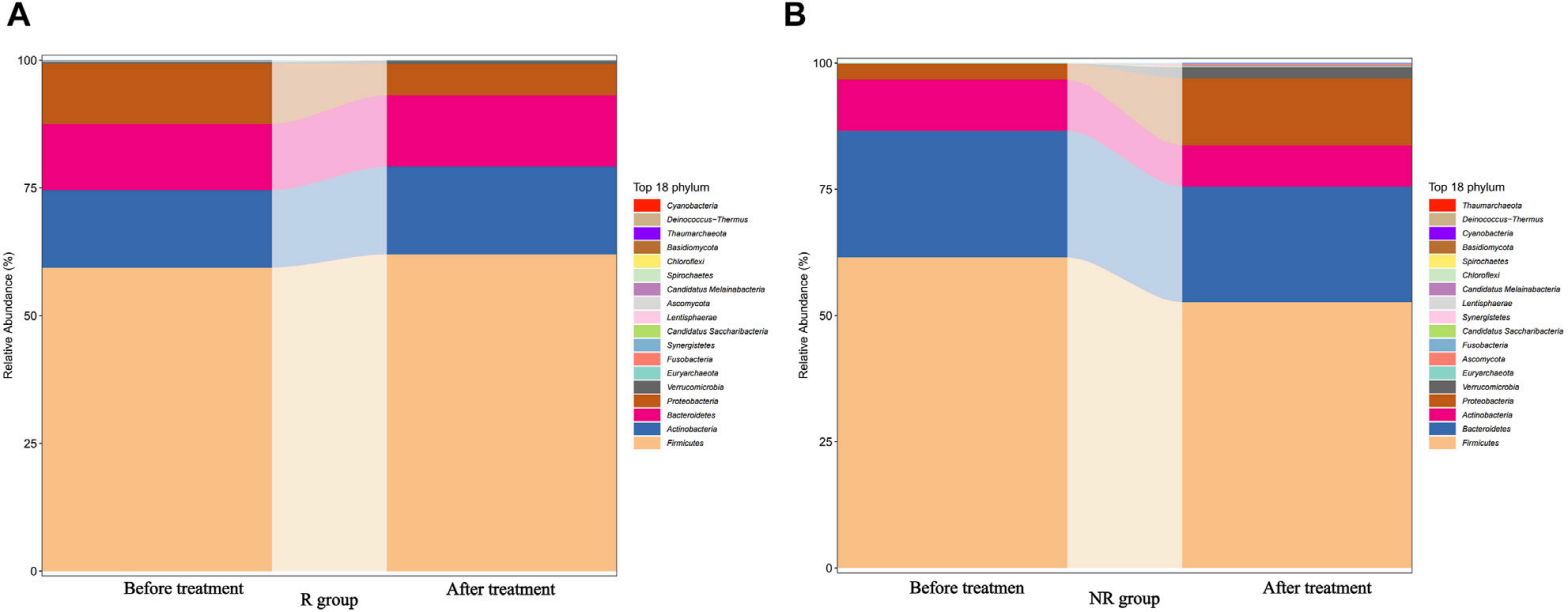
Changes in the gut microbiome composition of R **(A)** and NR **(B)** groups during treatment before and after treatment.

We further analyzed the compositional of the gut microbiome in the R and NR groups (Figures 3A, B). The species composition of the two groups was similar before and after treatment and was relatively stable during treatment (Figure 4).

### Association of treatment response with gut microbial diversity

**FIGURE 5:**
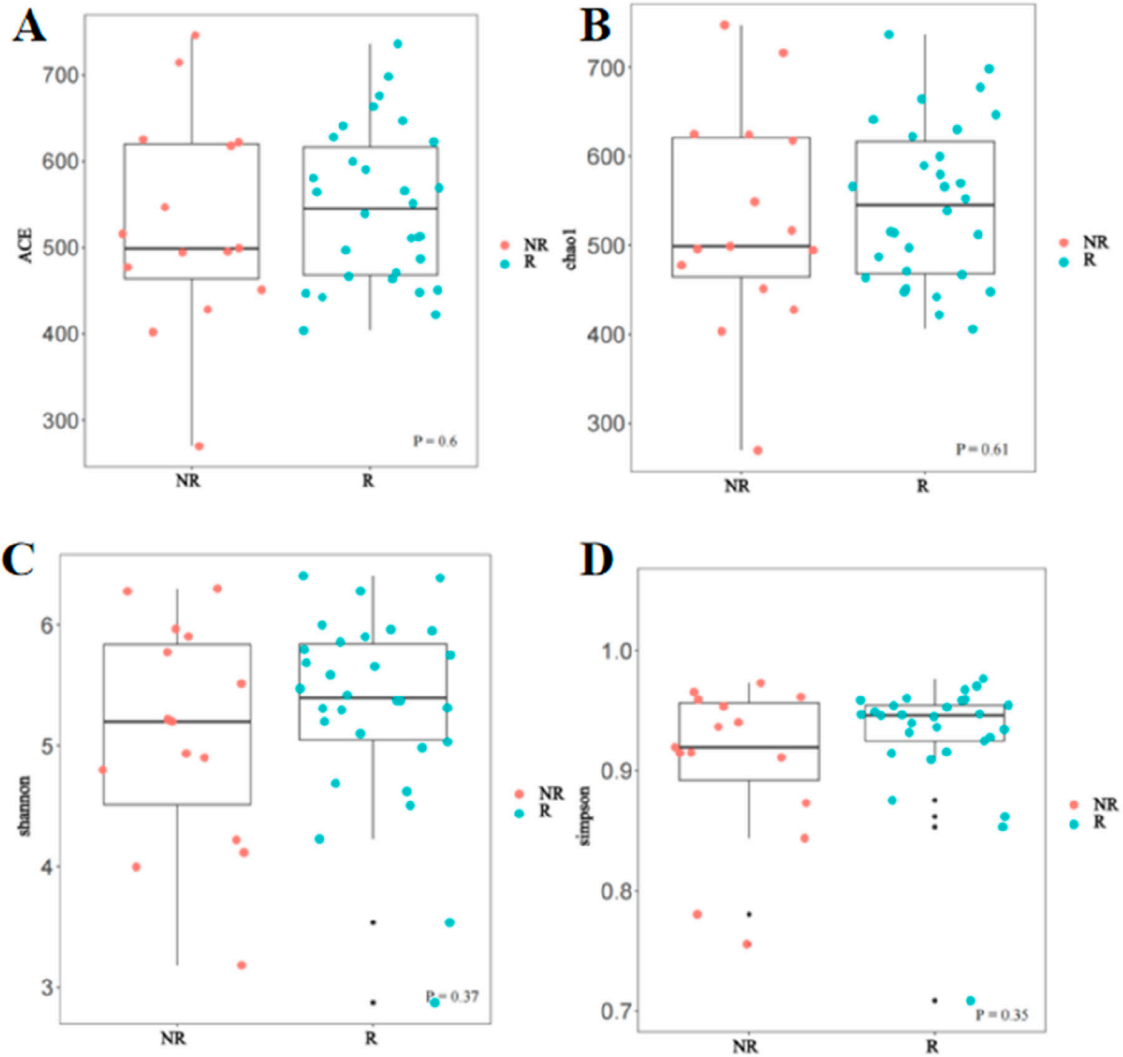
Comparison of Alpha diversity between the R and NR groups **(A)** ACE index; **(B)** Chao1 index; **(C)** Shannon index; **(D)** Simpson index.

In this study, Alpha and Beta diversity analyses were used to compare gut microbial diversity and richness of the fecal sample. There was no significant difference in Alpha diversity between the R group and the NR group. As shown in Figure 5, there was no statistical difference in the Chao1 index, ACE index, Shannon index, and Simpson index between the two groups.

**FIGURE 6:**
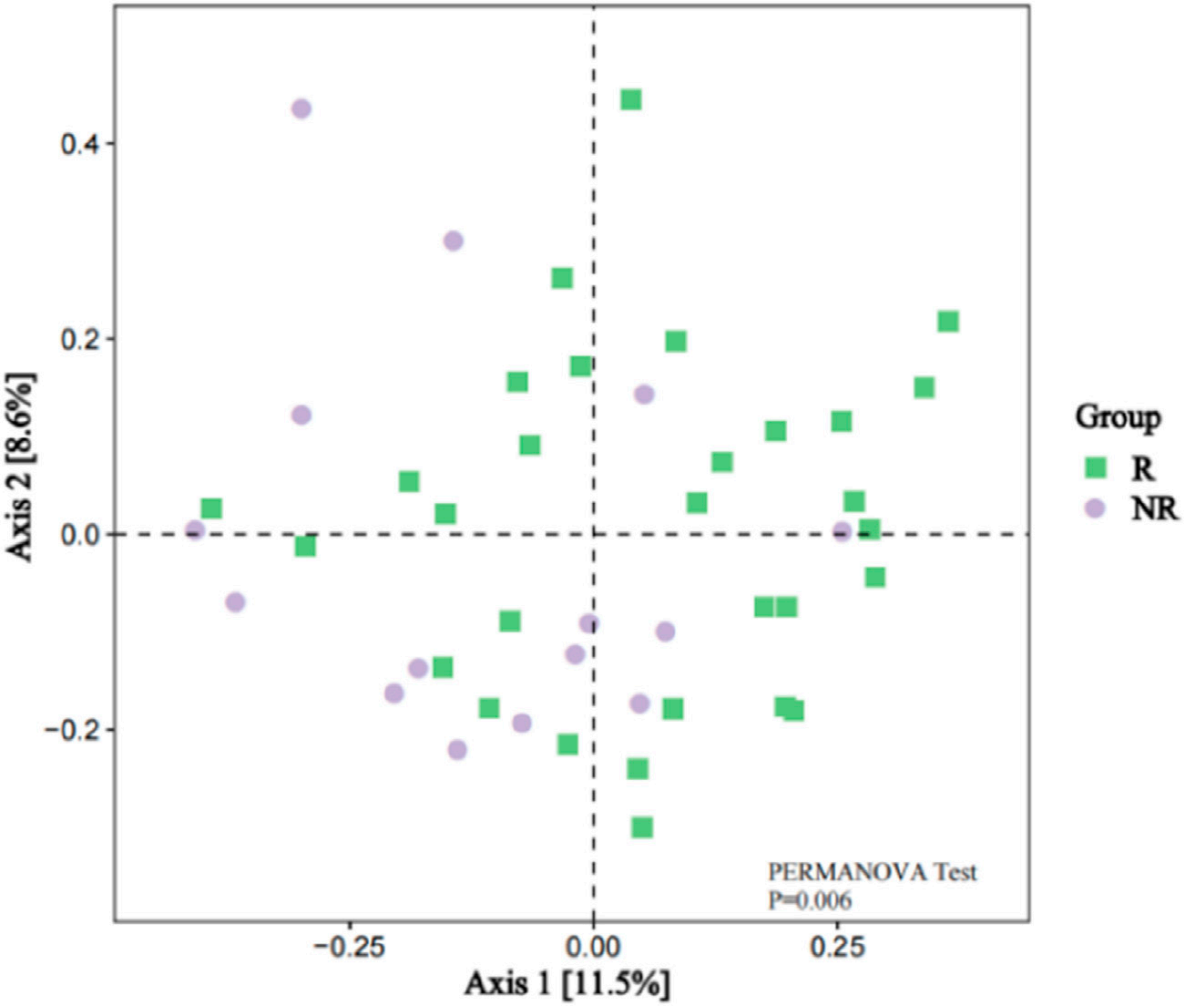
Beta Diversity between the R and NR groups (PCoA analysis).

However, the beta diversity evaluated by Bray-Curtis distance showed that patients in the R group were separated from those patients in the NR group by PCoA (Figure 6, PERMANOVA tests, P = 0.006).

### Differentially enriched bacterial taxa between R and NR groups

**FIGURE 7:**
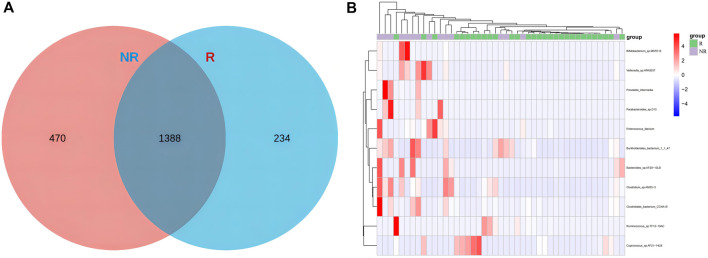
Venn diagram of species **(A)** and heat map of differential species **(B)** for R and NR groups.

Common and unique bacterial taxa between the two groups were analyzed. Venn diagram (Figure7A) showed that at the specie level, the R group and NR group shared 1,388 common species and there were 234 and 470 unique species in the R group and NR group, respectively. We then compared the abundance differences of species levels between samples and found a different cluster distribution of different species between the R and NR groups (Figure 7B).

**FIGURE 8:**
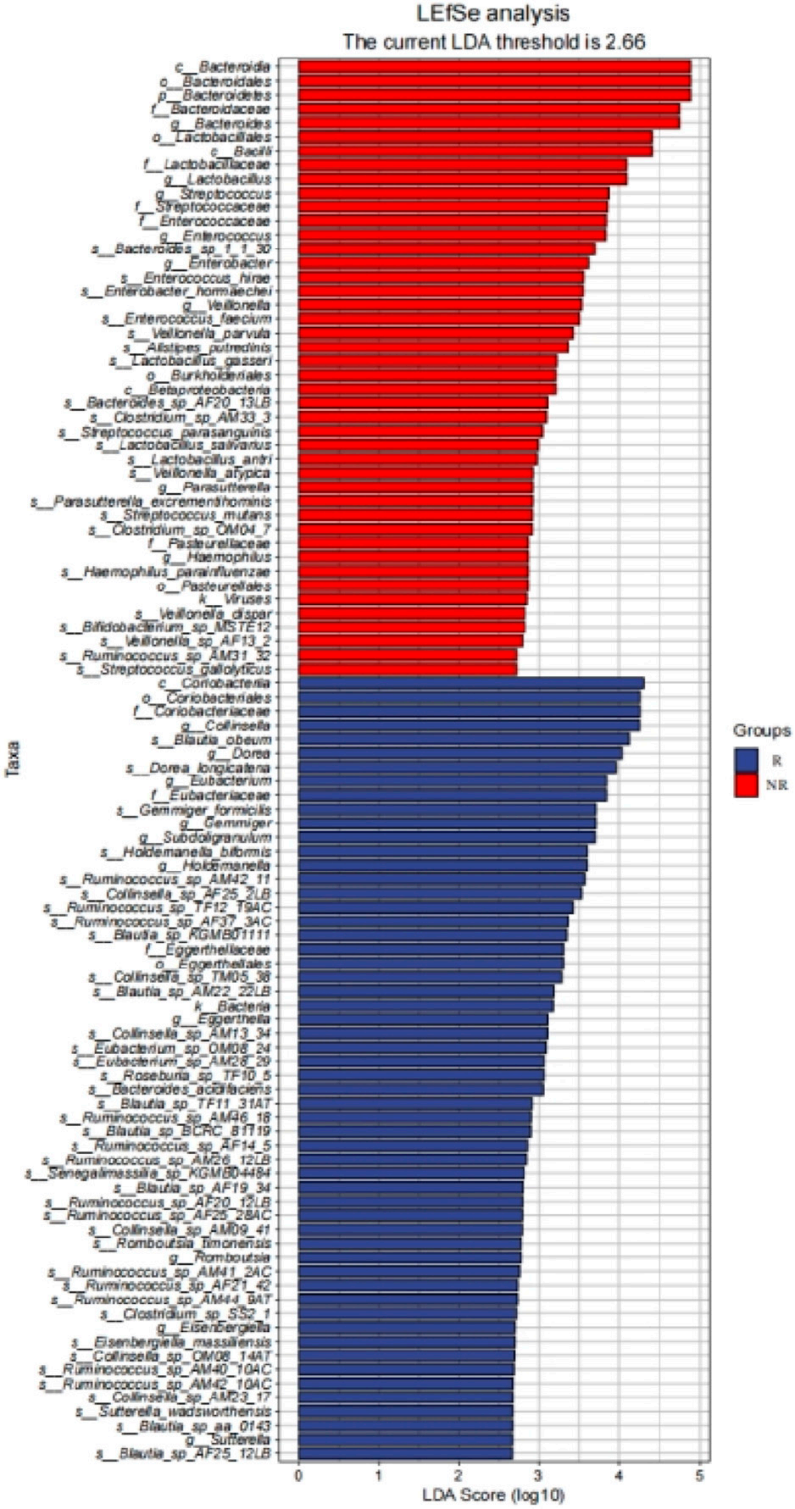
LEfSe identified significantly differentially bacterial taxa in the R group and NR group (LDA>2.66, p< 0.05).

We further identified 56 enriched bacterial taxa in the R group and 44 enriched bacterial taxa in the NR group based on the LEfSe analysis (LDA >2.66, P < 0.05) (Figure 8). Of note, Coriobacteriia class, Coriobacteriales order, Coriobacteriaceae family, and Collinsella genus were the top enriched taxa in the R group, while Bacteroidia class, Bacteroidales order, Bacteroidetes phylum, Bacteroidaceae family, *Bacteroides* genus were the top enriched bacterial taxa in the NR group (LDA >3, P < 0.05). Our results indicated that enrichment of specific bacterial taxa in gut microbiota was correlated with treatment response and that significantly enriched differential bacterial taxa could be used as potential biomarkers to predict the efficacy of HCC patients receiving anti-PD-1-based combination therapy.

### Association of survival benefit with enriched differential bacterial taxa

**FIGURE 9:**
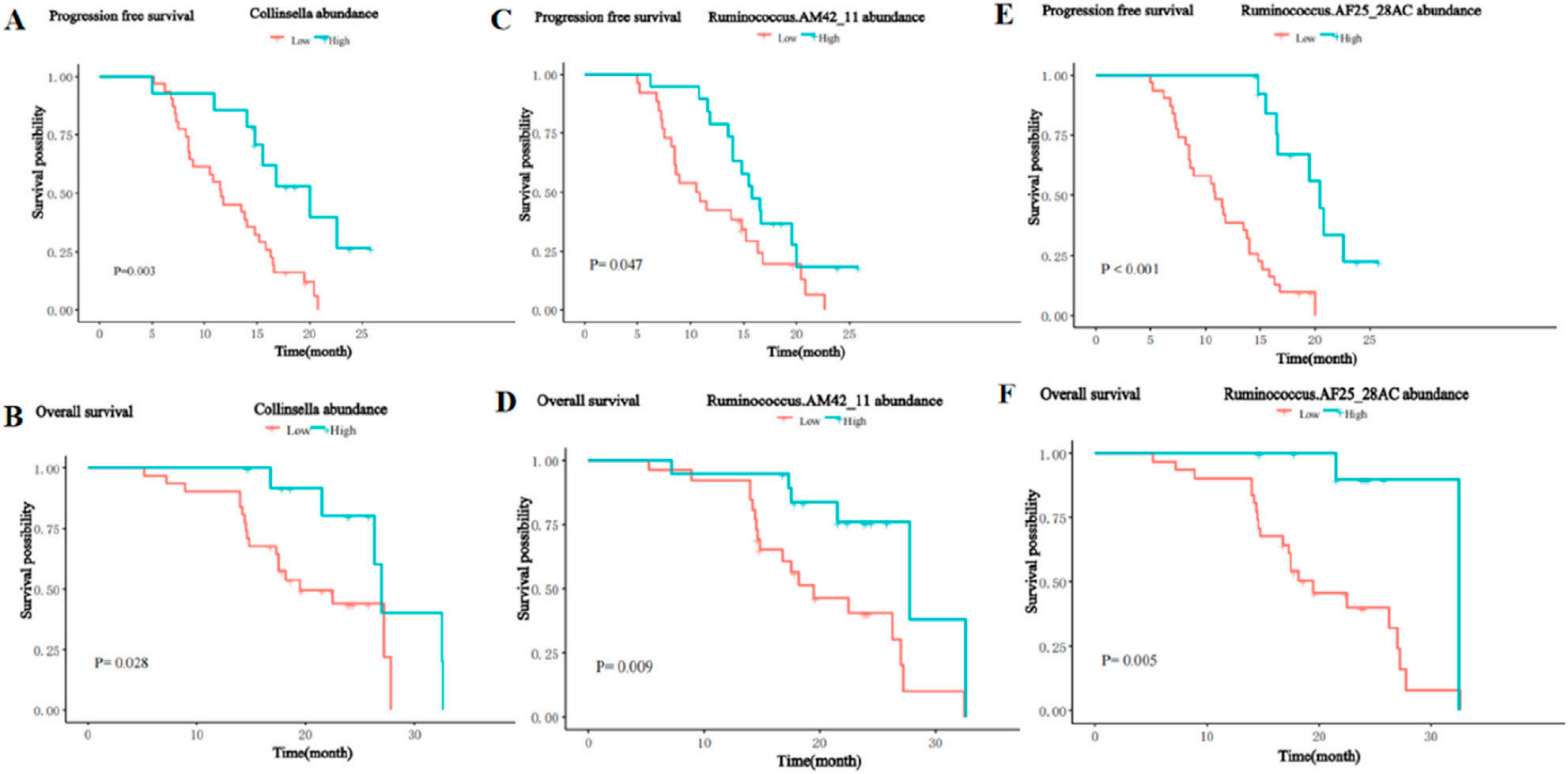
Kaplan-Meier curves for PFS and OS of patients with high and low abundance groups of significantly enriched bacterial taxa in the R group. **(A)** The PFS in the high and low abundance of Collinsella in the R group. **(B)** The OS in the high and low abundance of Collinsella in the R group. **(C)** The PFS in the high and low abundance of Ruminococcus_AM42_11 in the R group. **(D)** The OS in the high and low abundance of Ruminococcus_AM42_11 in the R group. **(E)** The PFS in the high and low abundance of Ruminococcus_AF25_28 in the R group. **(F)** The OS in the high and low abundance of Ruminococcus_AF25_28 in the R group.

To further analyze the impact of significantly enriched differential on the survival of patients, we stratified the 45 patients with HCC into a high abundance group and a low abundance group based on the mean abundance of these differentially enriched bacterial taxa in the R group and NR group. Survival analysis showed that the presence of Collinsella genus, Ruminococcus_AM42_11, and Ruminococcus_AF25_28 was significantly positively associated with PFS and OS. Patients with a higher abundance of Collinsella achieved longer PFS (median PFS (m PFS): 20.0 vs. 11.6 months, P = 0.003, (Figure 9A), and OS (median OS (m OS): 27.0 vs. 19.5 months, P = 0.028, Figure 9B) than patients with a lower abundance. The enrichment of Ruminococcus_AM42_11 and Ruminococcus_AF25_28 was also associated with better PFS (m PFS: 15.8 vs. 10.7 months, P = 0.047, Figure 9C; m PFS: 20.4 vs. 10.9 months, P< 0.001; Figure 9E) and OS (m OS: 27.8 vs. 19.5 months, p = 0.009, Figure 9D; m OS: 32.5 vs. 19.5 months, p = 0.005; Figure 9F).

**FIGURE 10:**
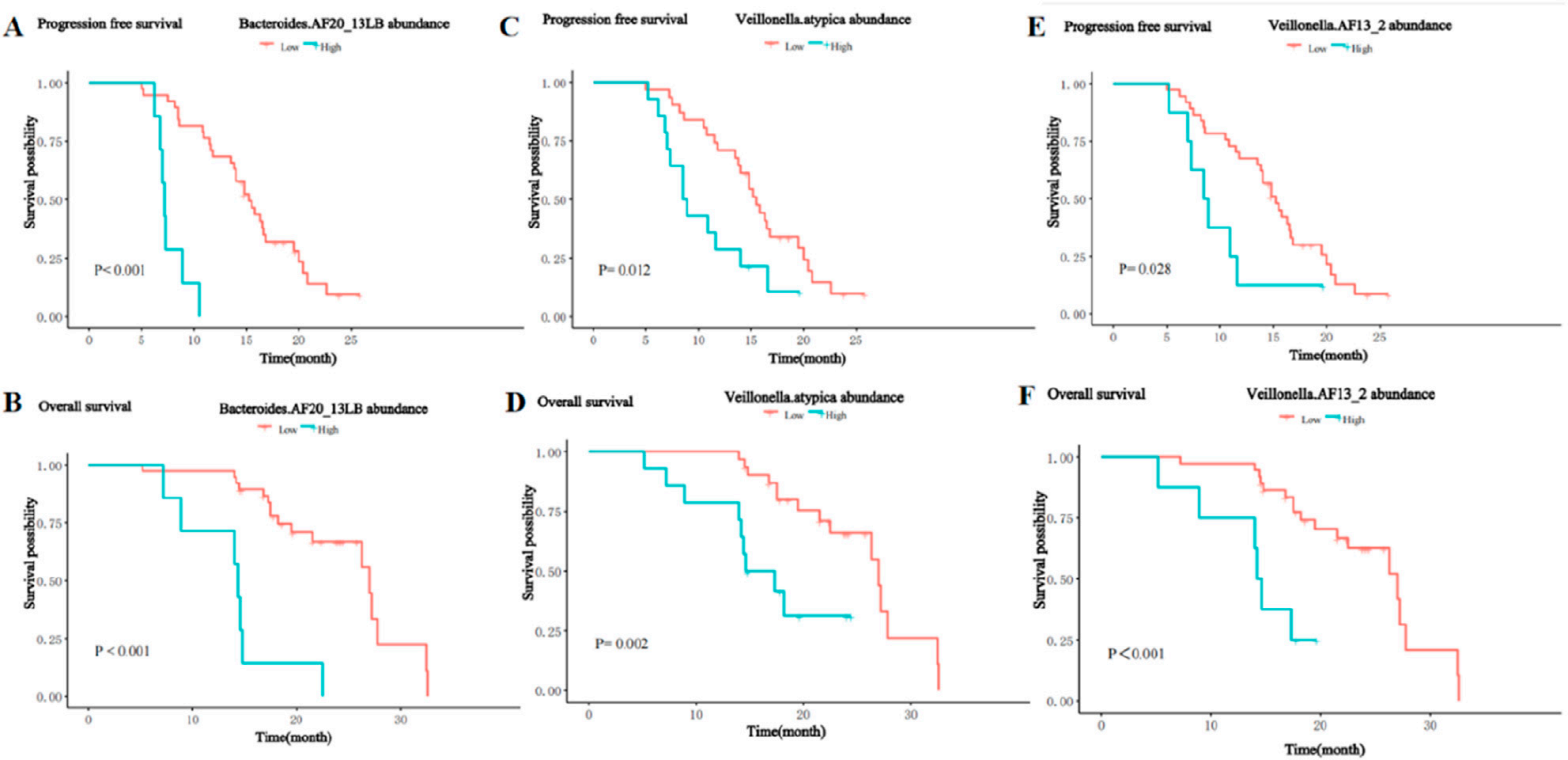
Kaplan-Meier curves for PFS and OS of patients with high and low abundance groups of significantly enriched bacterial taxa in the NR group. **(A)** The PFS in the high and low abundance of Bacteroides_AF20_13LB in the NR group. **(B)** The OS in the high and low abundance of Bacteroides_AF20_13LB in the NR group. **(C)** The PFS in the high and low abundance of Veillonella_atypic in the NR group. **(D)** The OS in the high and low abundance of Veillonella_atypic in the NR group. **(E)** The PFS in the high and low abundance of Veillonella_AF13_2 in the NR group. **(F)** The OS in the high and low abundance of Veillonella_AF13_2 in the NR group.

In contrast, the presence of the *Bacteroides*_AF20_13LB, Veillonella_atypica, and Veillonella_AF13_2 was significantly negatively associated with PFS and OS. Patients with a higher abundance of *Bacteroides*_AF20_13LB achieved shorter PFS (m PFS: 7.2 vs. 15.2 months, P< 0.001, Figure 10A) and OS (m OS: 14.4 vs. 27.0 months, P < 0.001, Figure 10B) than patients with a lower abundance. The enrichment of Veillonella_atypic and Veillonella_AF13_2 enrichment was also associated with shorter PFS (m PFS: 8.7 vs. 15.5 months, P = 0.012, Figure 10C; m PFS: 8.7 months vs. 15.2 months, P = 0.028; Figure 10E) and OS (m OS: 16.0 vs. 27.0 months, P = 0.002, Figure 10D; m OS: 14.4 vs. 27.0 months, P < 0.001; Figure 10F).

### Univariate and multivariate analysis of PFS and OS

The results of the univariate analysis showed age, BCLC stage, largest tumor size, tumor distribution, extrahepatic metastasis, the abundance of Collinsella genus, Ruminococcus_AM42_11, *Bacteroides*_AF20_13LB, Veillonella_atypica, Veillonella_AF13_2, and Ruminococcus_AF25_28AC were related factors for PFS. The multivariate analysis showed that the abundance of *Bacteroides*_AF20_13LB, Ruminococcus_ AF25_28AC, BCLC stage, and largest tumor size were independent related factors for PFS (Table 2).

**TABLE 2:** Univariate and multivariate analysis of progression-free survival.

Factor	Progression-free survival			
	Univariate analysis		Multivariate analysis	
	HR (95%CI)	P value	HR (95%CI)	P value
Sex (Femela/Male)	0.524 (0.208–1.317)	0.143		
Age (≥60/<60 years)	2.398 (1.234–4.658)	0.012	2.411 (0.797–7.293)	0.119
ECOG (1/0)	2.903 (0.867–9.714)	0.129		
Cirrhosis (Yes/No)	1.102 (0.576–2.107)	0.770		
Child-Pugh Grade (A/B)	0.976 (0.296–3.217)	0.968		
BCLC stage (C/B)	4.249 (1.408–12.826)	0.004	3.622 (1.111–11.805)	0.033
Largest tumor size (≥10/<10 cm)	4.318 (2.035–9.162)	<0.001	7.034 (2.313–21.397)	0.001
Tumor distribution (Single lobe/double lobe)	2.204 (1.07–4.538)	0.025	2.531 (0.928–6.903)	0.070
PVTT (Yes/No)	1.917 (0.943–3.896)	0.072		
Extrahepatic metastasis (Yes/No)	2.428 (1.178–5.006)	0.020	1.556 (0.481–5.037)	0.461
AFP (<400/≥400 ng/mL)	1.173 (0.564–2.443)	0.666		
AST (≥40/<40 U/L)	1.948 (0.942–4.029)	0.063		
ALT (≥40/<40 U/L)	1.133 (0.589–2.181)	0.708		
Collinsella abundance (High/Low)	0.294 (0.127–0.681)	0.002	0.499 (0.152–1.644)	0.254
Ruminococcus.AM42_11 abundance (High/Low)	0.514 (0.263–1.005)	0.048	0.597 (0.221–1.617)	0.310
*Bacteroides*.AF20_13LB abundance (High/Low)	11.175 (3.768–33.14)	<0.001	10.255 (1.908–55.130)	0.007
Veillonella.atypica abundance (High/Low)	2.457 (1.193–5.059)	0.020	2.177 (0.896–5.291)	0.086
Veillonella.AF13_2 abundance (High/Low)	2.53 (1.077–5.944)	0.051		
Ruminococcus.AF25_28AC abundance (High/Low)	0.145 (0.055–0.383)	<0.001	0.180 (0.051–0.634)	0.008

Abbreviations: ECOG-PS, eastern cooperative oncology group performance status; BCLC, barcelona clinic liver cancer; AFP, alpha-fetoprotein; ALT, alanine aminotransferase; AST, aspartate aminotransferase; PVTT, portal vein tumor thrombus.

In addition, univariate analysis showed age, ECOG, largest tumor size, BCLC stage, PVTT, extrahepatic metastasis, the abundance of Collinsella genus, Ruminococcus_AM42_11, *Bacteroides*_AF20_13LB, Veillonella_atypica, Veillonella_AF13_2, and Ruminococcus_AF25_28AC were related factors of OS. Multivariate analysis showed that the abundance of *Bacteroides*_AF20_13LB was an independent related factor of OS (Table 3).

**TABLE 3:** Univariate and multivariate analysis of overall survival.

Factor	Overall survival			
	Univariate analysis		Multivariate analysis	
	HR (95%CI)	P value	HR (95%CI)	P value
Sex (Femela/Male)	0.566 (0.165–1.939)	0.333		
Age (≥60/<60 years)	2.373 (0.998–5.641)	0.048	0.904 (0.1569–5.213)	0.910
ECOG (1/0)	4.456 (1.266–15.687)	0.048	3.105 (0.425–22.680)	0.264
Cirrhosis (Yes/No)	1.328 (0.574–3.069)	0.506		
Child-Pugh Grade (A/B)	1.335 (0.381–4.679)	0.662		
BCLC stage (C/B)	4.249 (1.408–12.826)	0.004	1.808 (0.0781–41.852)	0.712
Largest tumor size (≥10/<10 cm)	2.457 (1.05–5.749)	0.039	4.919 (0.882–27.417)	0.069
Tumor distribution (Single lobe/double lobe)	0.957 (0.395–2.317)	0.922		
PVTT (Yes/No)	4.075 (1.554–10.683)	0.002	2.488 (0.302–20.497)	0.397
Extrahepatic metastasis (Yes/No)	2.715 (1.073–6.87)	0.039	8.074 (0.924–70.570)	0.059
AFP (<400/≥400 ng/mL)	2.179 (0.718–6.608)	0.139		
AST (≥40/<40 U/L)	1.864 (0.72–4.828)	0.182		
ALT (≥40/<40 U/L)	0.788 (0.331–1.878)	0.591		
Collinsella abundance (High/Low)	0.307 (0.102–0.926)	0.020	0.854 (0.170–4.285)	0.848
Ruminococcus_AM42_11 abundance (High/Low)	0.285 (0.105–0.772)	0.007	0.327 (0.070–1.519)	0.154
*Bacteroides*_AF20_13LB abundance (High/Low)	8.16 (3.042–21.883)	<0.001	20.858 (1.302–334.079)	0.032
Veillonella_atypica abundance (High/Low)	3.919 (1.533–10.02)	0.006	2.568 (0.354–18.646)	0.351
Veillonella_AF13_2 abundance (High/Low)	5.242 (1.863–14.749)	0.004	1.432 (0.184–11.130)	0.732
Ruminococcus_AF25_28AC abundance (High/Low)	0.16 (0.037–0.686)	0.002	0.599 (0.066–5.429)	0.649

Abbreviations: ECOG-PS, eastern cooperative oncology group performance status; BCLC, barcelona clinic liver cancer; AFP, alpha-fetoprotein; ALT, alanine aminotransferase; AST, aspartate aminotransferase; PVTT, portal vein tumor thrombus.

The above results indicated that significantly enriched microbial taxa were an independent related factor of PFS and OS that can influence the survival outcomes of patients.

## Discussion

The current study demonstrated that the enrichment of specific bacterial taxa in gut microbiota might enhance the clinical response and prolong the survival of HCC patients receiving anti-PD-1 combination therapy, which can predict the survival benefit for patients with HCC receiving anti-PD-1 combination therapy.

Immune checkpoint inhibitors combined with molecularly targeted drugs have become an important treatment modality for HCC. As a multi-targeted TKI, Lenvatinib can reverse the immunosuppressive microenvironment of HCC, and the combination therapy with PD-1 inhibitor can enhance the immune anti-tumor efficacy, showing superior anti-tumor effect and survival benefit than monotherapy (Adachi et al., 2022; Liu et al., 2019). Despite the tremendous advances in immune-combination therapy in unresectable HCC, treatment response varies widely among individual patients, and effective biomarkers for predicting immune-combination targeted therapy are still lacking. The gut microbiota can affect the antitumor immune response of cancer through innate and adaptive immunity, and increasing evidence indicates that the diversity of gut microbiota is related to the clinical efficacy of anti-PD-1/PD-L1 monotherapy in melanoma, non-small cell lung cancer, gastrointestinal tumors, and renal cell carcinoma, and may become potential biomarkers for the prediction of immunotherapy efficacy (Gopalakrishnan et al., 2018a; Zheng et al., 2019; Jin et al., 2019; Routy et al., 2018; Temraz et al., 2019). This study is the first to explore the correlation between gut microbes and the efficacy and survival of receiving PD-1 inhibitors in combination with lenvatinib for HCC. Our results demonstrated that significantly enriched microbial taxa can be a promising novel non-invasive biomarker for predicting survival in patients with HCC treated with immune combination therapy.

The gut microbiota is recognized as a crucial factor in HCC development and anticancer immune responses. A study cohort of 75 HBV-infected HCC patients from China demonstrated increased gut microbial diversity from cirrhosis to early HCC patients, and 30 gut microbes screened based on a random forest model achieved an area under the curve of 80.64% in identifying early HCC and non-HCC, demonstrating the strong diagnostic potential of gut microbial markers in HCC (Ren et al., 2019). In addition, a small number of clinical studies have demonstrated that the diversity of gut microbiota is strongly associated with response to immunotherapy for HCC (Mao et al., 2021; Lee et al., 2022). Our results found no statistical difference in Alpha diversity such as the Chao1index, ACE index, Shannon index, and Simpson index between the gut microbiome of patients in the R and NR groups, but Beta diversity analysis revealed significant differences in microbial bacteria diversity between the two groups and found that some of the significantly enriched microbial taxa were independently associated with patient survival. These results suggested that the diversity and abundance of gut microbes can affect the therapeutic efficacy and survival prognosis of HCC patients, which was consistent with the findings reported in previous studies. Furthermore, our findings suggested that the composition of the gut microbiome remains approximately stable during treatment and did not change before and after treatment so that the baseline gut microbiome characteristics can be used as a promising biomarker for HCC.

The composition and diversity of gut microbes can significantly impact the host’s immune system, and increasing evidence suggests that specific enriched microbial taxa may influence the antitumor response to ICI therapy in cancer patients (Gopalakrishnan et al., 2018b; Ting et al., 2022; Lu et al., 2022; Mao et al., 2021; Lee et al., 2022; Zheng et al., 2019). Our study found that there were significantly enriched microbial taxa between the two groups, and most of the significantly enriched bacteria taxa in the R group belonged to Clostridiales, such as Lachnospiraceae, Eubacteriaceae, Ruminococcus, etc., and most of the significantly enriched bacteria taxa in the NR group belonged to Bacteroidia. Some of the enriched bacteria taxa were consistent with the findings of gut microbial studies in other cancer species receiving immunotherapy. For example, studies reported that in patients with gastrointestinal tumors and metastatic melanoma treated by PD-1 inhibitors, most of the enriched bacteria taxa in the treatment response group belonged to Clostridiales, while *Bacteroides* was significantly enriched in the treatment non-response group (Zheng et al., 2019; Peng et al., 2020; Peters et al., 2019). Although the gut bacteria in published studies do not completely overlap with the treatment response in this study, these findings support that specific enriched bacteria taxa may influence response to ICI therapy. There has been clear evidence in previous preclinical models that the different compositions of the gut microbiota may change the immune response of PD-1 inhibitor therapy by affecting the tumor microenvironment. The presence of “beneficial” gut bacteria such as Ackermannia and Bifidobacterium can increase antigen presentation and improve the function of effector T cells in the peripheral and tumor microenvironment to enhance the local and systemic anti-tumor immune response (Iida et al., 2013; Davar et al., 2021). Conversely, the presence of “harmful” gut bacteria such as *Bacteroides* and *E. coli* can limit the infiltration of lymphocytes and myeloid cells in the tumor and impair antigen presentation, resulting in an impaired anti-tumor immune response (Singh et al., 2021; Temraz et al., 2021). These findings highlight the potential of the gut microbiota as a therapeutic target in patients undergoing immunotherapy.

This study further analyzed the correlation of significantly enriched microbial taxa with survival and found that patients with a high abundance of Collinsella and Ruminococcus achieved significantly longer PFS and OS, while patients with a high abundance of *Bacteroides* and Veillonella_atypica had significantly shorter PFS and OS. Some previous studies also found that Collinsella and Ruminococcus could enhance the host anti-tumor immune activity, improve the immunotherapy response, and prolong patient survival in patients with melanoma and liver cancer, which is consistent with our results (Hakozaki et al., 2020; Tanoue et al., 2019). Many animal and clinical trials have found that the use of antibiotics or fecal bacteria transplantation changes the composition of the gut microbe, which has proved that regulating the composition of the gut microbes can enhance the effectiveness of immunotherapy and reduce immune-related adverse effects, Therefore, the gut microbiota may increase the susceptibility of HCC cells to apoptosis induction by secreting modulators or producing metabolites, thereby increasing the immune antitumor response to PD-1 inhibitor. (Dzutsev et al., 2017). These findings suggest that changes in gut microbiota can suppress or promote the immune response, potentially enhancing the anti-tumor response to immunotherapy in patients with HCC and prolonging survival.

Our study still has some limitations. First of all, the significant enrichment of microbial taxa in our study with the enrichment of other cancer species does not completely overlap, this may be because the study of the different tumor types, dietary habits and regions, sample size, and other reasons can affect the composition and diversity of gut microbiota, the future needs to design a prospective multicenter randomized clinical trial to prove the results. Second, this study used the immune-combination regimen of Lenvatinib and PD-1 inhibitor, and the results are needed to confirm the first-line treatment regimen of Atezolizumab plus bevacizumab. Third, we could not determine the causal relationship between differential bacteria and tumor outcomes, so *in vitro* trials and clinical studies are needed to elucidate the biological mechanisms of gut microbiota-mediated immune regulation. Finally, the majority of the patients in this study were HBV-related HCC, and whether these findings can be generalized to non-viral HCC patients treated with ICIs requires further investigation.

In summary, the enrichment of specific gut microbiota affected clinical efficacy and survival benefits in HCC treated with anti-PD-1-based combination therapy and may be a promising non-invasive gut microbial biomarker and a new strategy for modulating immunotherapy in HCC.

## Data Availability

The data presented in the study are deposited in the NCBI SRA database, accession number PRJNA1142390.
